# Ultrasound-based breast nodule malignancy diagnosis using CNN enhanced by knowledge distillation

**DOI:** 10.1016/j.isci.2026.118030

**Published:** 2026-10-26

**Authors:** Tao Hu, Rongrong Ru, Yingying Ding, Vicky Yang Wang, Dong Xu

**Affiliations:** 1 Taizhou Key Laboratory of Minimally Invasive Interventional Therapy & Artificial Intelligence, Taizhou Campus of Zhejiang Cancer Hospital (Taizhou Cancer Hospital), Taizhou, Zhejiang 317502, China; 2 Affiliated Xiaoshan Hospital, Hangzhou Normal University, No.728 North Yucai Road, Hangzhou, Zhejiang 311202, China; 3 Center of Intelligent Diagnosis and Therapy (Taizhou), Zhejiang Cancer Hospital, Hangzhou Institute of Medicine (HIM), Chinese Academy of Sciences, Taizhou, Zhejiang 317502, China; 4 Wenling Institute of Big Data and Artificial Intelligence in Medicine, Taizhou, Zhejiang 317502, China; 5 Department of Diagnostic Ultrasound Imaging & Interventional Therapy, Zhejiang Cancer Hospital, Hangzhou Institute of Medicine (HIM), Chinese Academy of Sciences, Hangzhou, Zhejiang 310022, China; 6 Wenzhou Medical University, Wenzhou, Zhejiang 325035, China; 7 School of Life Science and Technology, Xi’an Jiaotong University, Xi’an, Shaanxi 710049, China

**Keywords:** breast ultrasound, deep learning, lesion classification, knowledge distillation, feature fusion

## Abstract

Accurate discrimination of benign and malignant breast lesions is essential for early diagnosis, yet remains challenging due to lesion heterogeneity and interobserver variability in ultrasound assessment. We developed a deep learning (DL) framework integrating dual inverted bottleneck blocks (Dual-IB), adaptive hierarchical feature fusion, and knowledge distillation (KD) for breast ultrasound lesion classification. The model was trained and evaluated on public datasets and validated on an independent external cohort to assess its generalizability. Results demonstrated superior performance compared with baseline methods and resident radiologists, while maintaining robust performance across diverse datasets and imaging conditions. On the external validation dataset, the model achieved the best performance, with an accuracy (Acc) of 92.64% and an F1-score of 92.37%. Furthermore, class activation mapping (CAM) visualizations revealed focused and anatomically plausible attention patterns within lesion regions, aiding interpretation and suggesting its potential to assist in breast ultrasound lesion classification.

## Highlights

• Teacher-student knowledge distillation transfers high-level semantic representations
• Targeted pruning and hierarchical feature fusion balance efficiency and performance
• A hybrid loss improves hard-sample learning and classification performance
• Activation maps show focused and anatomically plausible attention within lesions

## Graphical abstract

**Figure:**
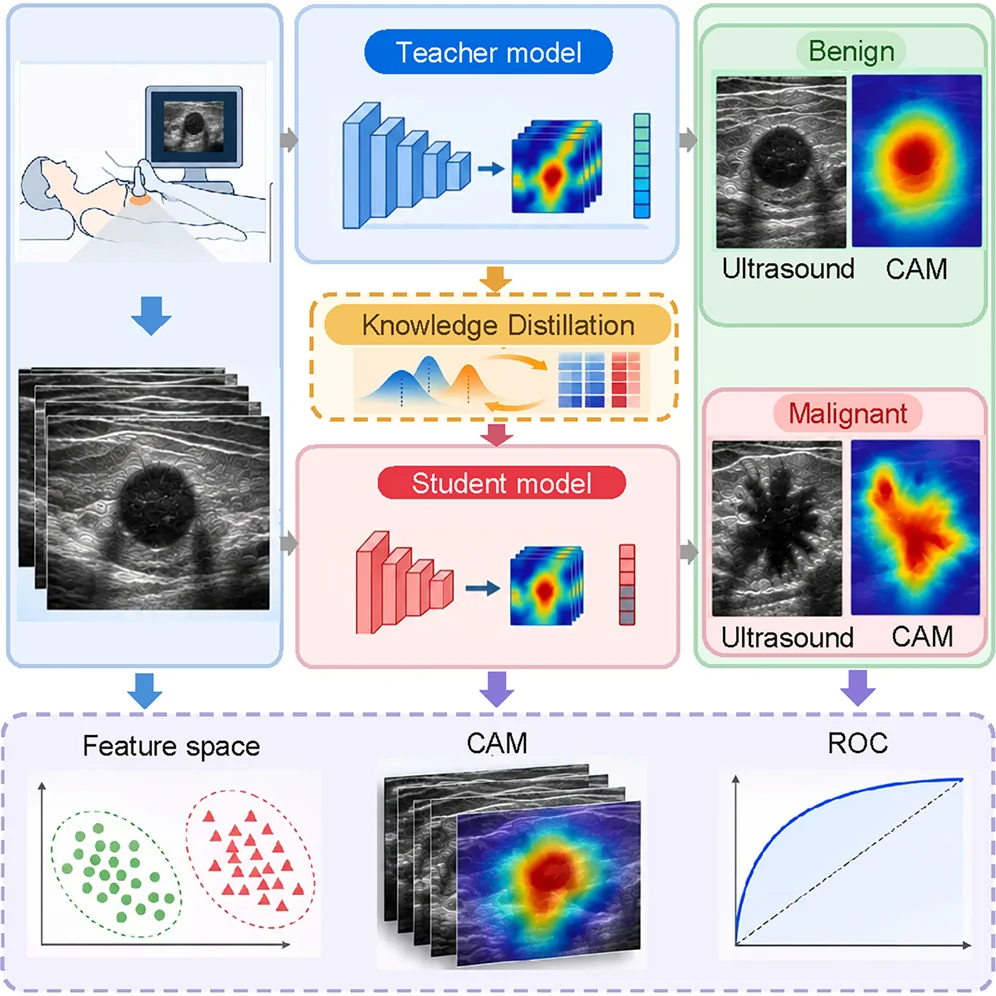

## Teaser

Oncology; Artificial intelligence applications

## Introduction

Breast cancer is one of the most prevalent malignant tumors among women worldwide and a major threat to women’s health and life safety.^1^^,^^2^^,^^3^ According to the latest statistical data from the World Health Organization (WHO) and the International Agency for Research on Cancer (IARC), the incidence rate of breast cancer among women globally has surpassed that of lung cancer, ranking first, and shows an increasing trend year by year.^4^^,^^5^ Early screening and accurate diagnosis are considered crucial links to reduce the mortality rate of breast cancer and improve the cure rate.^6^ Traditional breast imaging examination methods include mammography, ultrasound, magnetic resonance imaging (MRI), etc.^7^ Each of these examinations has its own advantages and disadvantages: Mammography can display microcalcifications in the breast but has poor performance in imaging dense breasts^8^; MRI can provide high-resolution breast images but is expensive and complex to operate^9^; ultrasound, as a safe, economical, and non-radiative imaging method, is widely used in the early detection and follow-up of breast cancer due to its excellent imaging capability for dense breast tissue.^10^ However, ultrasound images have inherent limitations, such as severe noise interference, limited resolution, blurred boundaries, and imaging quality that is easily affected by the operator’s experience,^11^^,^^12^ leading to strong subjectivity and uncertainty in distinguishing benign from malignant breast lesions.^13^ These issues seriously affect the accuracy (Acc) of early breast cancer identification and benign-malignant diagnosis, especially for BI-RADS category 4 nodules, whose malignant detection rate varies widely (2%–95%), posing significant challenges to clinical diagnosis.^14^^,^^15^

In recent years, with the development of artificial intelligence (AI), deep learning (DL), and radiomics technologies, significant progress has been made in research on AI-assisted diagnosis of breast cancer based on medical imaging. Convolutional neural network (CNN) can automatically learn high-dimensional features from breast ultrasound images through multi-layer nonlinear feature extraction networks, enabling the detection and classification of lesion regions, which has significantly improved the Acc and consistency of diagnosis.^16^ Researchers have successfully applied models such as ResNet,^17^ DarkNet,^18^ and EfficientNet^19^ to the task of benign-malignant diagnosis of breast ultrasound images, and some results have reached or even exceeded the level of experienced radiologists.^20^ The Transformer architecture has also achieved breakthrough progress in the field of computer vision, and its self-attention mechanism can capture global dependency information and enhance feature expression capabilities.^21^ Networks based on vision transformer (ViT) and Swin Transformer have demonstrated comparable or even superior performance to CNN in the classification and segmentation of breast ultrasound images.^22^ Transformers can effectively integrate local and global information, showing significant advantages for tumor regions with complex morphologies and blurred boundaries. However, transformer models usually have large computational complexity, high requirements for data volume, and poor performance on small amounts of labeled data.

In ultrasound imaging research, Ra et al.^23^ enhanced radiomics features using a large language model for mammography-based breast tumor classification, achieving 67.1% Acc on the VinDr-Mammo dataset and 83.9% Acc on the INbreast dataset. Elshenawy et al.^24^ conducted a comparative analysis for federated learning approaches for multi-class classification in ultrasound imaging, achieving a best average Acc of 73.31% and a macro-F1 score of 67.3% using the FedProx-MobileNet framework despite data heterogeneity. Ma et al. proposed DFMFI,^25^ a dynamic fusion multi-scale feature interaction model for ultrasound breast cancer detection, achieving 87.12% Acc and 91.56% AUC, outperforming state-of-the-art hybrid models. El-Shazli et al.^26^ proposed the MSAE-DL framework, which integrates hybrid self-attention mechanisms with ensemble classification for digital breast tomosynthesis, achieving an Acc of 90.13%. Focusing on feature integration, Wang et al.^27^ proposed CTMF-Net, a CNN-transformer multi-stage fusion network for breast ultrasound classification, achieving 90.14% Acc on SYSU and 92.04% Acc on UDIAT, outperforming state-of-the-art methods. Addressing the need for interpretability, Naas et al.^28^ developed an explainable ViT-based CAD system for histopathological breast cancer classification on the BreakHis dataset, achieving up to 92.0% Acc across different magnification levels. Rahman et al.^29^ introduced HyFormer-Net, a synergistic CNN-Transformer model for ultrasound images, which utilizes interpretable multi-scale fusion to reach an Acc of 93.2%. Similarly, Yu et al.^30^ developed BiaCanDet, a space-time attention neural network analyzing bioelectrical impedance data, reporting an Acc of 93.37%. Tang et al.^31^ proposed a multi-stage attention architecture based on nonlinear spiking neural P systems with autapses for breast cancer histopathology classification, achieving 96.32% Acc on the BreakHis dataset across all magnification levels. Cho et al.^32^ further enhanced segmentation and classification using an attention-based fusion network on multi-modal ultrasound images, achieving a classification Acc of 98.46%. Kabir et al.^33^ proposed a CWC-MP-MC image representation with an optimized ViT for breast ultrasound tumor classification, achieving over 98% Acc across the Mendeley, UDIAT, and BUSI datasets. Singh et al.^34^ presented MammXAI, an adaptive multi-model approach integrating XAI with multi-modality images, which achieved a remarkable Acc of 99.6%. Kiran et al.^35^ advanced breast ultrasound diagnostics by combining EfficientNetB3 with k-Nearest Neighbors, achieving 100% Acc along with perfect precision, recall, and F1-score.

Despite the significant progress made in DL-based classification methods for breast ultrasound images in recent years, existing studies still face several limitations and challenges.^36^ First, traditional CNNs primarily rely on local convolution operations for feature extraction. Although they perform well in capturing local textures and edge information, their ability to model global semantic information and long-range dependencies remains limited. Second, large-scale CNN models often involve a substantial number of parameters. While they can achieve high classification Acc in controlled experimental settings, their performance may degrade when dealing with small-sample or challenging lesion cases.^37^ Therefore, effectively enhancing global feature representation while improving robustness and discriminative capability remains a key challenge in breast ultrasound image classification.

This study proposes a benign-malignant classification method for breast ultrasound images based on CNN and knowledge distillation (KD) strategies, aiming to achieve model efficiency and interpretability while maintaining high diagnostic Acc. Specifically, we design a CNN architecture that integrates model pruning, dual inverted bottleneck blocks (Dual-IB), and an adaptive hierarchical feature fusion module. This design enables the efficient fusion of local texture information and global semantic cues, realizing an accurate and robust representation of breast mass morphology. Furthermore, the incorporation of a KD mechanism allows the transfer of deep feature representations from a high-performance teacher model to a student model, significantly enhancing the student model’s classification performance and generalization ability. Additionally, a hybrid LMF loss function is introduced to enhance the discriminative capability for hard-to-classify samples, effectively improving the model’s recognition performance for challenging cases.

## Results

### Hyperparameter tuning of KD

The effects of the temperature parameter (T) and the distillation weight (α) on the proposed KD framework were systematically investigated based on 5-fold cross-validation. The corresponding results are summarized in Table 1, where all performance metrics are reported as the mean ± standard deviation across the 5-folds. Both hyperparameters were found to significantly affect the classification performance. Among all evaluated configurations, T = 6 and α = 0.5 consistently achieved the best overall performance on both the BUSI and UDIAT datasets. When the temperature was too low, the teacher outputs contained limited dark knowledge, whereas an excessively high temperature over-smoothed the probability distribution and reduced the discriminative capability of the student model. Similarly, decreasing α below 0.5 caused the student network to rely excessively on the teacher’s soft targets, leading to performance degradation. Overall, both datasets exhibited a consistent trend of performance first improving and then declining as T and α increased. Therefore, T = 6 and α = 0.5 were selected as the optimal hyperparameter configuration for all subsequent experiments.

**Table 1:** Experimental results with different distillation parameters

Dataset	T	α	Acc (%)	Pr (%)	Re (%)	F1 (%)
BUSI	4	0.5	94.31 ± 0.89	93.96 ± 2.27	93.12 ± 1.89	93.53 ± 2.06
	4	0.6	94.68 ± 0.75	94.72 ± 1.31	93.06 ± 1.15	93.83 ± 1.37
	6	0.5	95.07 ± 2.04	94.82 ± 1.34	94.21 ± 2.35	94.50 ± 1.46
	6	0.6	94.51 ± 0.94	94.05 ± 1.98	93.68 ± 1.51	93.85 ± 1.64
	8	0.6	94.37 ± 2.56	94.55 ± 0.84	92.62 ± 1.17	93.54 ± 0.81
	8	0.7	93.76 ± 0.86	93.61 ± 1.98	92.41 ± 1.33	92.99 ± 0.28
UDIAT	4	0.5	95.57 ± 1.40	94.98 ± 1.72	94.86 ± 2.33	94.91 ± 0.93
	4	0.6	97.28 ± 0.52	97.46 ± 1.99	96.56 ± 0.91	96.99 ± 1.04
	6	0.5	97.59 ± 2.16	97.17 ± 2.56	97.52 ± 0.89	97.24 ± 1.27
	6	0.6	95.76 ± 0.57	95.61 ± 1.89	94.86 ± 1.72	95.23 ± 0.93
	8	0.6	95.16 ± 0.59	95.10 ± 1.33	93.93 ± 0.64	94.48 ± 2.18
	8	0.7	94.81 ± 1.77	94.62 ± 1.78	93.67 ± 1.35	94.11 ± 0.94

After determining the optimal KD hyperparameters (T = 6 and α = 0.5) from the experiments summarized in Table 1, these settings were fixed for all subsequent experiments. The influence of different teacher architectures was then investigated, and the comparison results are presented in Table 2. Among the evaluated teacher networks, DenseNet121 consistently achieved the best classification performance on both the BUSI and UDIAT datasets, outperforming ResNet50, EfficientNet-B0, and VGG16 across all evaluation metrics. This result indicates that the dense connectivity of DenseNet121 enables more effective feature reuse and richer supervisory information, thereby facilitating more efficient knowledge transfer to the lightweight student network. Although ResNet50 and EfficientNet-B0 also produced competitive performance, they remained slightly inferior to DenseNet121, while VGG16 yielded the weakest overall results. Therefore, DenseNet121 was selected as the teacher model for all subsequent experiments.

**Table 2:** Comparison of different teacher networks

Dataset	Teacher	Acc (%)	Pr (%)	Re (%)	F1 (%)
BUSI	ResNet50	94.26 ± 2.31	93.88 ± 1.72	93.54 ± 2.58	93.69 ± 1.86
	DenseNet121	95.07 ± 2.04	94.82 ± 1.34	94.21 ± 2.35	94.50 ± 1.46
	EfficientNet-B0	94.63 ± 1.91	94.27 ± 1.56	93.86 ± 2.12	94.04 ± 1.63
	VGG16	93.71 ± 2.67	93.24 ± 2.09	92.81 ± 2.74	93.01 ± 2.18
UDIAT	ResNet50	96.91 ± 2.42	96.58 ± 2.63	96.77 ± 1.41	96.64 ± 1.75
	DenseNet121	97.59 ± 2.16	97.17 ± 2.56	97.52 ± 0.89	97.24 ± 1.27
	EfficientNet-B0	97.18 ± 1.87	96.94 ± 2.21	97.09 ± 1.08	96.98 ± 1.43
	VGG16	96.35 ± 2.74	95.92 ± 2.81	96.16 ± 1.62	96.01 ± 0.91

To qualitatively evaluate the learned feature representations, t-SNE was employed to visualize the feature embeddings, as shown in Figure 1. Benign and malignant samples from both the BUSI and UDIAT datasets form compact and well-separated clusters with minimal overlap, demonstrating the strong discriminative capability of the proposed model. Although the UDIAT dataset exhibits slightly greater intra-class variation, the two classes remain clearly distinguishable. These qualitative results are consistent with the quantitative findings in Tables 1 and 2, further confirming the effectiveness of the proposed KD framework.

**Figure 1:**
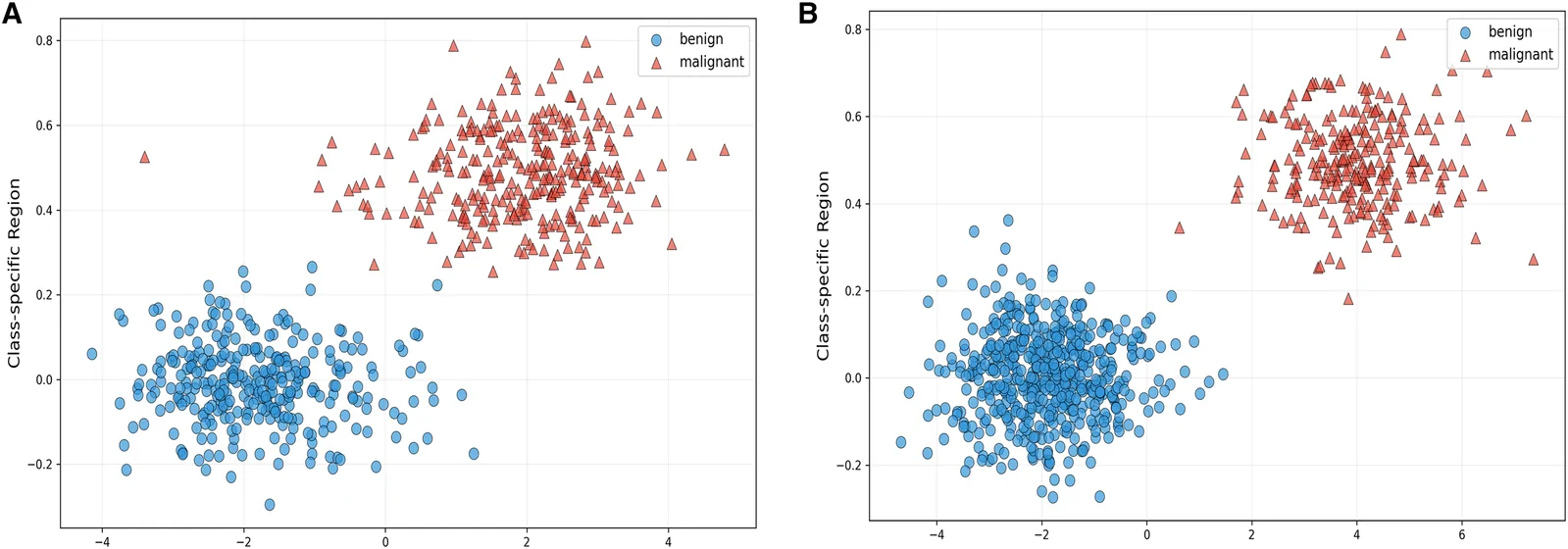
t-SNE visualization of learned feature representations on the breast ultrasound datasets (A) BUSI dataset. (B) UDIAT dataset.

### Ablation study

The ablation results are presented in Table 3, and the corresponding performance gains over the baseline are summarized in Table 4. As more modules were progressively incorporated into the baseline model, the classification performance consistently improved on both the BUSI and UDIAT datasets, demonstrating that each proposed component contributes positively to feature representation learning. In particular, the complete model achieved an Acc of 95.07% on the BUSI dataset and 97.59% on the UDIAT dataset, outperforming the baseline by 2.49% and 3.23%, respectively. The combination of Dual-IB, LMF, and HF produced complementary improvements, while the incorporation of KD further enhanced the student model, yielding the highest gains across all evaluation metrics. These results confirm the effectiveness of each individual module as well as their synergistic contribution to the proposed framework.

**Table 3:** Ablation study results of individual modules

Dataset	Model	Acc (%)	Pr (%)	Re (%)	F1 (%)
BUSI	baseline	92.58 ± 2.47	92.16 ± 1.83	91.74 ± 2.76	91.95 ± 2.12
	+Dual-IB	93.01 ± 2.15	92.73 ± 2.36	92.18 ± 1.68	92.45 ± 1.94
	+LMF	93.32 ± 1.58	93.03 ± 2.08	92.57 ± 2.42	92.81 ± 1.37
	+Dual-IB+ LMF	93.89 ± 2.03	93.61 ± 1.49	93.12 ± 2.21	93.37 ± 1.74
	+Dual-IB+ LMF +HF	94.28 ± 1.26	94.12 ± 2.14	93.72 ± 1.81	93.92 ± 0.98
	+Dual-IB+ LMF + HF+ KD	95.07 ± 2.04	94.82 ± 1.34	94.21 ± 2.35	94.50 ± 1.46
UDIAT	baseline	94.36 ± 2.68	94.02 ± 2.11	94.28 ± 1.76	94.15 ± 2.34
	+Dual-IB	94.81 ± 1.97	94.56 ± 2.53	94.73 ± 1.34	94.64 ± 2.08
	+LMF	95.07 ± 2.39	94.86 ± 1.62	95.03 ± 2.05	94.94 ± 1.28
	+Dual-IB+ LMF	95.86 ± 1.43	95.58 ± 2.27	95.69 ± 1.17	95.75 ± 1.96
	+Dual-IB+ LMF +HF	96.21 ± 2.18	95.98 ± 1.38	96.16 ± 1.73	96.07 ± 1.09
	+Dual-IB+ LMF + HF+ KD	97.59 ± 2.16	97.17 ± 2.56	97.52 ± 0.89	97.24 ± 1.27

**Table 4:** Performance differences between ablation modules and the baseline

Dataset	Model	ΔAcc	ΔPr	Δ Re	Δ F1
BUSI	baseline	–	–	–	–
	+Dual-IB	0.43	0.57	0.44	0.50
	+LMF	0.74	0.87	0.83	0.86
	+Dual-IB+ LMF	1.31	1.45	1.38	1.42
	+Dual-IB+ LMF +HF	1.70	1.96	1.98	1.97
	+Dual-IB+ LMF + HF+ KD	2.49	2.66	2.47	2.55
UDIAT	baseline	–	–	–	–
	+Dual-IB	0.45	0.54	0.45	0.49
	+LMF	0.71	0.84	0.75	0.79
	+Dual-IB+ LMF	1.50	1.56	1.41	1.60
	+Dual-IB+ LMF +HF	1.85	1.96	1.88	1.92
	+Dual-IB+ LMF + HF+ KD	3.23	3.15	3.24	3.09

To conduct an interpretability analysis of the proposed model, we adopted multiple mainstream class activation mapping (CAM) methods for feature visualization of breast ultrasound images, including GradCAM, GradCAM++, XGradCAM, EigenGradCAM, and LayerCAM, as shown in Figure 2. These methods allow for intuitive observation of the key regions focused on by the model in the images, thereby verifying the reliability of the model when distinguishing breast lesion categories. GradCAM and GradCAM++ can clearly highlight lesion regions, but their responses to edge details are relatively vague. Compared with GradCAM, GradCAM++ is more sensitive to small yet critical lesion points. While maintaining the overall regional response, XGradCAM can enhance the gradient weights of key features, making the lesion contours focused on by the model more accurate. EigenGradCAM provides responses based on the principal components of features, which can highlight the most significant abnormal regions in the images and help understand the model’s judgment basis for the overall structure. LayerCAM, on the other hand, can perform weighted fusion on feature maps from different convolutional layers, achieving multi-scale attention to lesion regions and ensuring that subtle lesions also receive significant responses.

**Figure 2:**
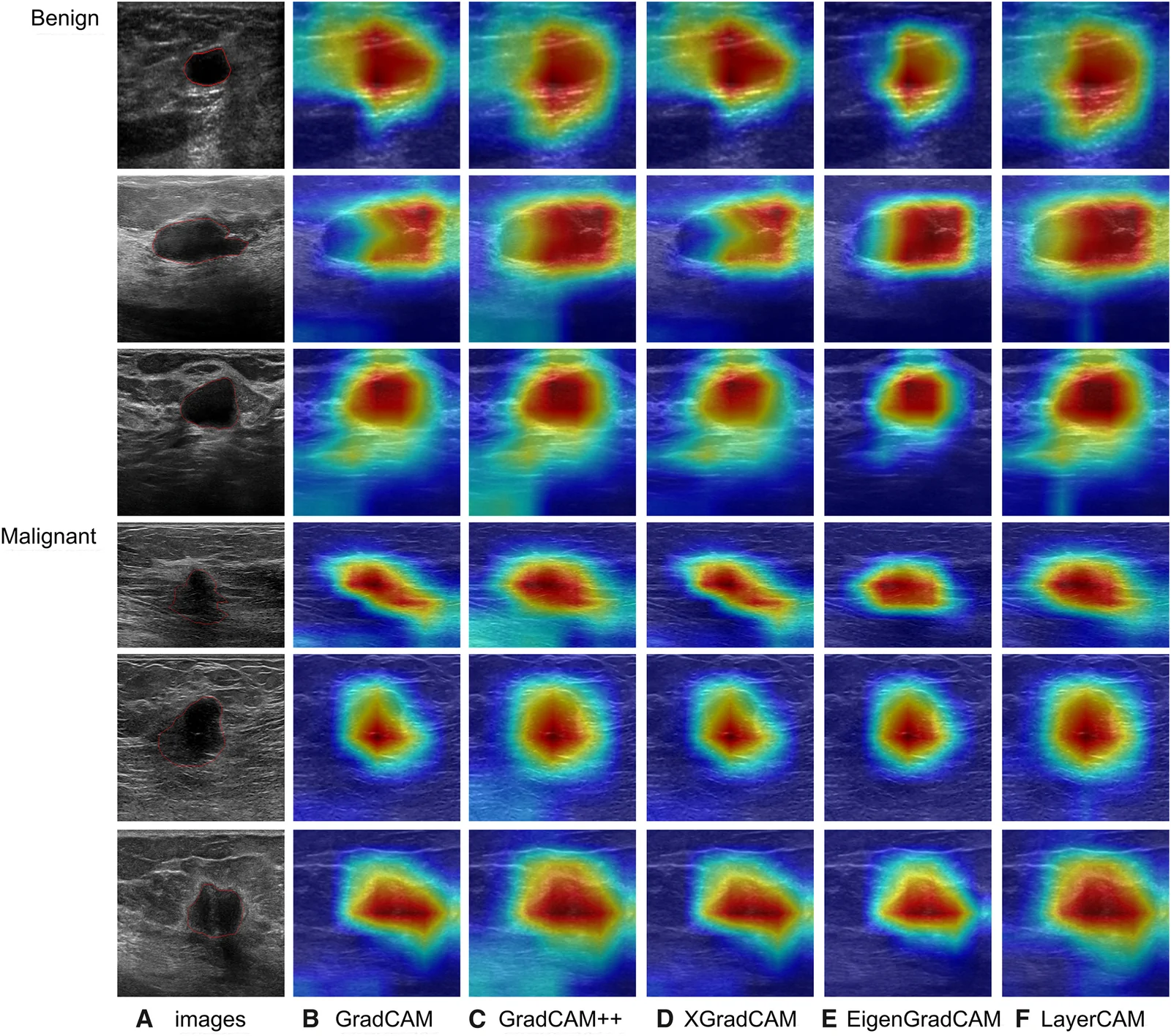
Feature visualization of breast ultrasound images using various CAM methods (A) Original image. (B) GradCAM. (C) GradCAM++. (D) XGradCAM. (E) EigenGradCAM. (F) LayerCAM.

As can be observed from the aforementioned visualization analysis, the proposed model can focus on the key regions of lesions in breast ultrasound images, rather than background noise or irrelevant tissue regions. This verifies that the model not only achieves high-Acc classification but also possesses favorable interpretability.

### Comparison with state-of-the-art methods

The performance comparison between the proposed method and state-of-the-art approaches under 5-fold cross-validation is presented in Table 5. The proposed model consistently achieved the best performance on both the BUSI and UDIAT datasets across all evaluation metrics. On the BUSI dataset, the proposed method obtained an Acc of 95.07% ± 2.04%, F1-score of 94.50% ± 1.46%, and AUC of 0.979 ± 0.009, outperforming all CNN- and transformer-based baseline models. On the UDIAT dataset, the proposed method further improved the performance, achieving an Acc of 97.59% ± 2.16%, F1-score of 97.24% ± 1.27%, and AUC of 0.988 ± 0.014. Compared with the strongest baseline model ConvNeXt, the proposed method achieved improvements of 1.32% in Acc, 1.42% in F1-score, and 0.006 in AUC on the UDIAT dataset. The consistent improvements across different datasets and evaluation metrics demonstrate that the proposed KD framework effectively enhances feature representation and improves the discriminative capability of the model.

**Table 5:** Performance comparison under 5-fold cross-validation

Dataset	Model	Acc (%)	Pr (%)	Re (%)	F1 (%)	AUC
BUSI	DarkNet	91.64 ± 2.31	90.48 ± 2.08	90.92 ± 2.47	90.70 ± 1.86	0.942 ± 0.021
	ShuffleNetV2	92.83 ± 1.74	91.81 ± 2.26	92.05 ± 1.53	91.93 ± 1.88	0.953 ± 0.008
	ResNet18	93.52 ± 2.14	93.08 ± 1.47	92.14 ± 2.35	92.61 ± 1.71	0.960 ± 0.014
	MobilenetV3	93.76 ± 1.39	93.31 ± 2.18	92.47 ± 1.72	92.89 ± 0.77	0.963 ± 0.012
	MobilenetV4	94.11 ± 2.02	93.84 ± 1.56	92.88 ± 2.11	93.36 ± 1.43	0.966 ± 0.007
	Swin-Transformer	94.62 ± 1.18	94.83 ± 2.27	93.08 ± 1.34	93.95 ± 1.62	0.976 ± 0.023
	Vit	94.25 ± 2.43	93.92 ± 1.82	93.01 ± 2.06	93.46 ± 1.57	0.969 ± 0.012
	ConvNeXt	94.46 ± 1.63	94.31 ± 1.29	93.07 ± 2.24	93.68 ± 1.46	0.973 ± 0.013
	Ours	95.07 ± 2.04	94.82 ± 1.34	94.21 ± 2.35	94.50 ± 1.46	0.979 ± 0.009
UDIAT	DarkNet	92.84 ± 2.58	91.96 ± 2.14	92.08 ± 2.67	92.02 ± 1.95	0.951 ± 0.024
	ShuffleNetV2	94.08 ± 1.81	94.63 ± 2.42	91.98 ± 1.56	93.29 ± 2.03	0.958 ± 0.007
	ResNet18	94.53 ± 2.21	94.46 ± 1.68	92.96 ± 2.18	93.70 ± 1.54	0.966 ± 0.011
	MobilenetV3	94.91 ± 1.42	95.26 ± 2.37	93.21 ± 1.63	94.22 ± 0.93	0.969 ± 0.014
	MobilenetV4	95.48 ± 2.09	95.19 ± 1.84	94.46 ± 2.74	94.82 ± 1.39	0.976 ± 0.008
	Swin-Transformer	96.03 ± 1.27	95.44 ± 2.08	95.69 ± 1.31	95.56 ± 1.58	0.978 ± 0.022
	Vit	94.88 ± 2.36	94.53 ± 1.73	93.81 ± 2.12	94.17 ± 1.47	0.973 ± 0.013
	ConvNeXt	96.27 ± 1.54	96.32 ± 1.21	95.33 ± 2.29	95.82 ± 1.32	0.982 ± 0.007
	Ours	97.59 ± 2.16	97.17 ± 2.56	97.52 ± 0.89	97.24 ± 1.27	0.988 ± 0.014

To further assess the generalization ability of the proposed model, external validation was performed on an independent private dataset that was not used during model training, and the results are summarized in Table 6. As shown in Table 6, the proposed model achieved the best performance, with an accuracy of 92.64%, precision of 92.56%, recall of 92.18%, F1-score of 92.37%, and AUC of 0.956. Compared with the other methods, our model consistently improved all evaluation metrics, demonstrating stronger feature representation and robustness under unseen data distributions. These results confirm the superior generalization capability and potential clinical applicability of the proposed approach. Figure 3 presents the confusion matrix of the proposed method on the external validation dataset, which provides a visual confirmation of its excellent classification performance and demonstrates accurate discrimination between benign and malignant lesions with limited misclassification. Despite the distribution differences between the public and private datasets, the proposed method maintained consistently superior classification performance, demonstrating its robustness and strong generalization ability. These findings further highlight the potential of the proposed framework for practical clinical applications in breast ultrasound image classification.

**Table 6:** Performance comparison on the independent private dataset for external validation

Model	Acc (%)	Pr (%)	Re (%)	F1 (%)	AUC
DarkNet	88.56	87.41	88.02	87.71	0.928
ShuffleNetV2	89.67	88.78	89.08	88.93	0.934
ResNet18	90.31	90.02	89.33	89.67	0.939
MobilenetV3	90.87	90.61	89.96	90.28	0.944
MobilenetV4	90.63	90.18	89.74	89.96	0.942
Swin-Transformer	91.54	91.72	90.36	91.03	0.952
Vit	91.22	90.73	90.28	90.50	0.946
ConvNeXt	91.08	91.15	90.47	90.81	0.947
Ours	92.64	92.56	92.18	92.37	0.956

**Figure 3:**
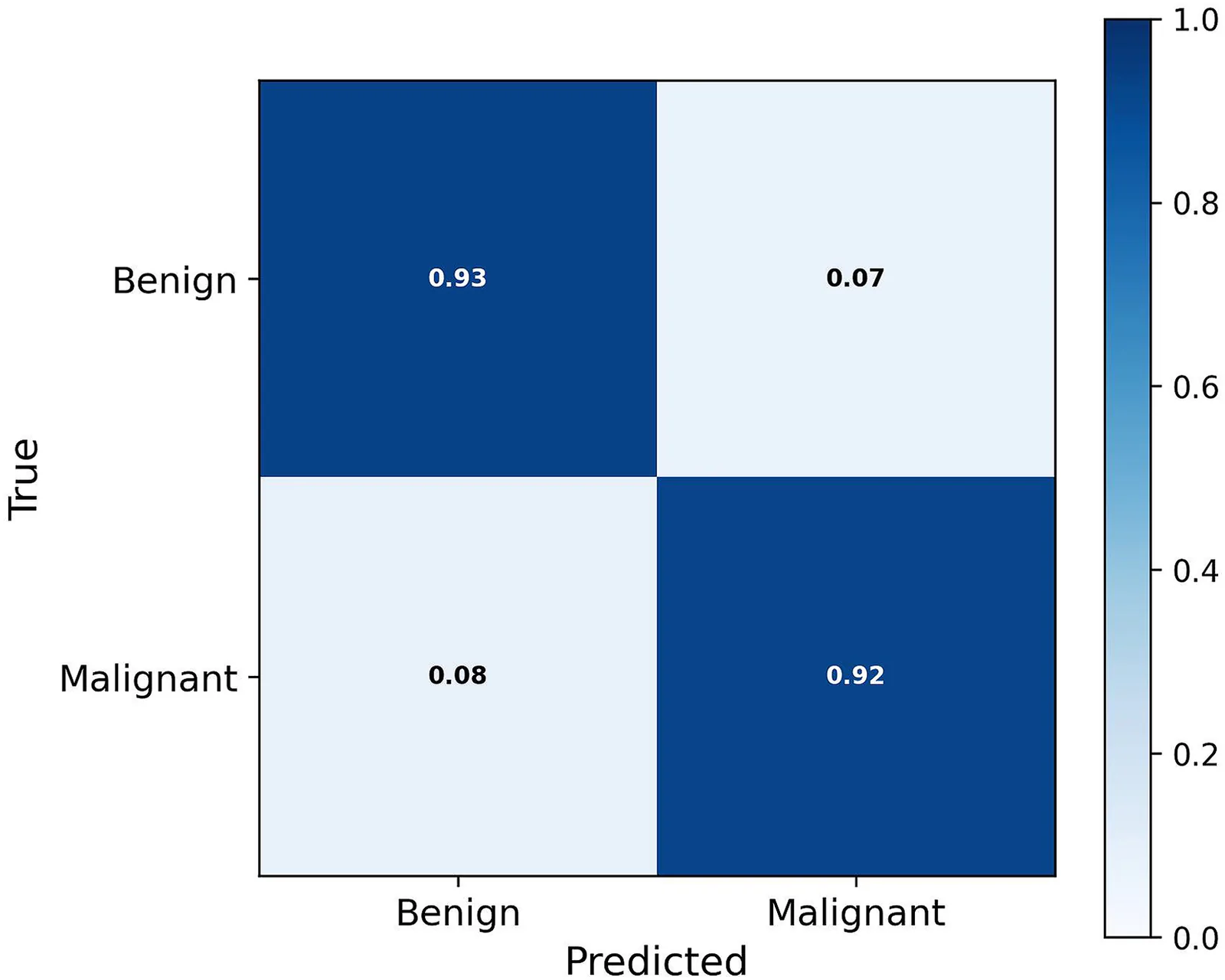
Confusion matrix on the external validation

The ROC curves of the proposed method and the competing models on the external validation dataset are presented in Figure 4. The proposed method achieved the highest AUC of 0.956, outperforming all comparison models, while the strongest baseline, Swin-Transformer, achieved an AUC of 0.952. Furthermore, the ROC curve of the proposed method consistently remained above those of the competing models across most operating points, indicating superior discriminative capability and a better balance between sensitivity and specificity. These results further confirm the robustness and generalization ability of the proposed framework on unseen clinical data. Table 7 presents a comparison of diagnostic performance between the proposed model and radiologists with different levels of experience, including two residents, two attending physicians, and two chief physicians. The results show that diagnostic Acc improved with increasing clinical experience, with chief physicians achieving higher Acc than attending physicians and residents. The proposed model achieved the highest diagnostic Acc of 92.64%, exceeding the accuracies of residents at 68.39%, attending physicians at 76.14%, and chief physicians at 83.90%.

**Figure 4:**
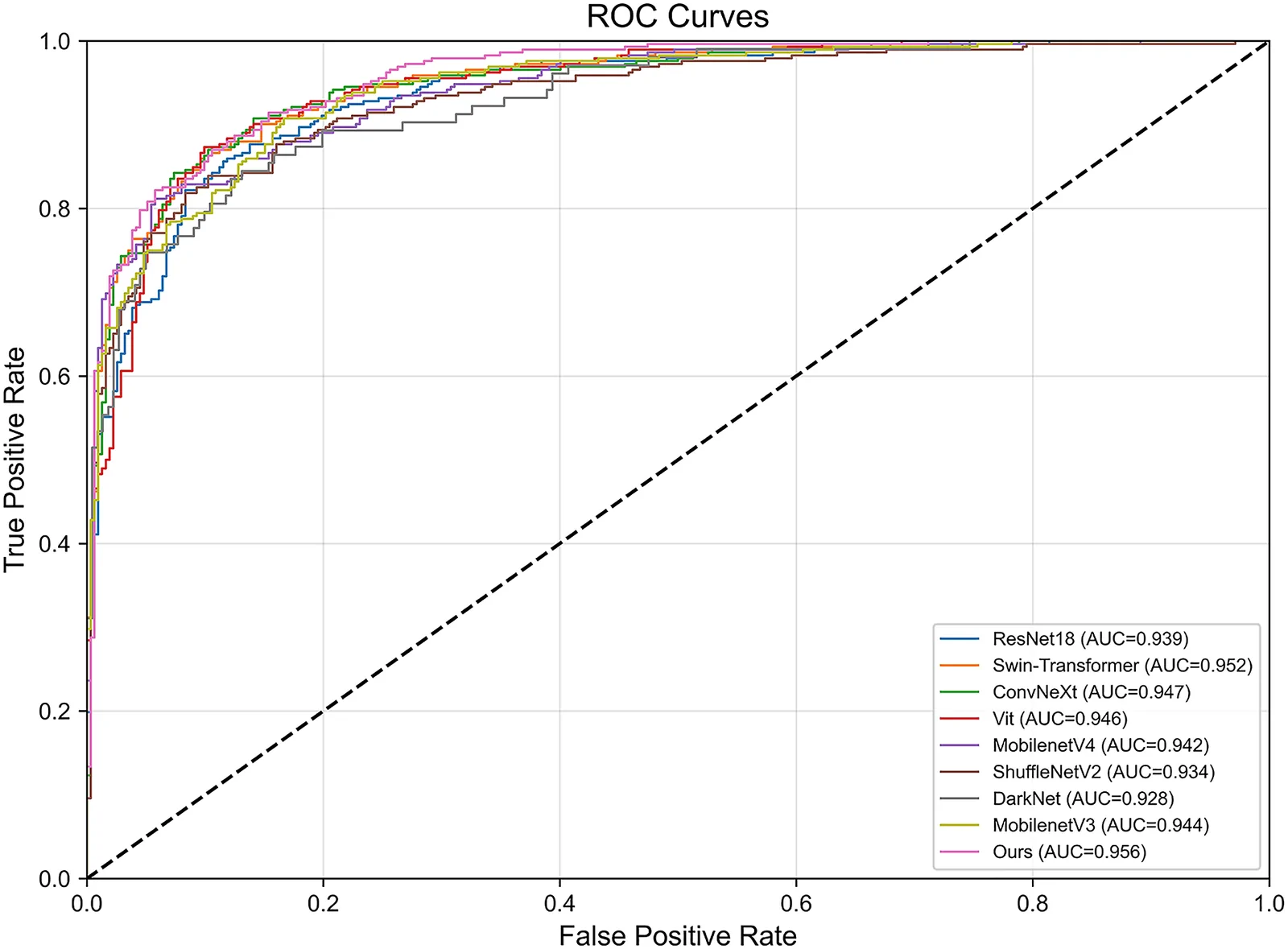
ROC curves of different models on private dataset

**Table 7:** Diagnostic performance comparison between the proposed model and radiologists

Model	Acc (%)	Pr (%)	Re (%)	F1 (%)
Residents	68.39	67.80	65.84	66.80
Attending physicians	76.14	75.95	74.07	75.00
Chief physicians	83.90	83.47	83.13	83.30
Ours	92.64	92.56	92.18	92.37

Considering that lesion size and image contrast are two important factors affecting the diagnostic difficulty of breast ultrasound images, the model performance was evaluated across different lesion sizes and contrast levels. Table 8 presents a quantitative subgroup analysis on the private external validation dataset. The proposed model achieved an overall Acc of 92.64% and an F1-score of 92.37%. However, its performance declined in more challenging cases. Specifically, for small lesions measuring ≤10 mm, the Acc and F1-score decreased to 87.14% and 86.67%, respectively. A similar trend was observed for low-contrast images, where the Acc and F1-score were 88.10% and 87.72%, respectively. Low-contrast images were defined by experienced breast ultrasound physicians based on lesion-to-background visibility. In contrast, the model performed better on lesions larger than 10 mm and on medium- to high-contrast images, achieving accuracies of 93.53% and 94.93%, respectively. The poorest performance was observed in cases where lesions were both small and low-contrast, with Acc and F1-score decreasing to 80.65% and 79.31%, respectively.

**Table 8:** Quantitative subgroup analysis on the private external validation dataset

Subgroup	Num	Acc (%)	Pr (%)	Re (%)	F1 (%)
Overall private dataset	503	92.64	92.56	92.18	92.37
Small lesions ≤10 mm	70	87.14	88.24	85.71	86.67
Lesions >10 mm	433	93.53	93.25	93.91	93.58
Low-contrast images	168	88.10	88.89	86.59	87.72
Medium/high-contrast images	335	94.93	94.51	95.12	94.81
Small and low-contrast lesions	31	80.65	82.14	76.67	79.31

These results demonstrate that the proposed model not only possesses stronger discriminative power but also exhibits excellent robustness and generalization ability across different breast ultrasound datasets, including real-world private data, confirming its effectiveness for breast lesion classification tasks. Figure 5 compares feature localization of ResNet18, MobileNetV3, Swin-Transformer, and the proposed method on breast ultrasound images. ResNet18 shows dispersed attention with partial focus on irrelevant regions, while MobileNetV3 captures lesions more clearly but sometimes incompletely. Swin-Transformer provides more consistent global attention, though boundaries and small lesions remain imprecise. In contrast, the proposed method produces concentrated activation over entire lesions, achieving more accurate feature representation and superior localization, which underlies its improved classification performance. Furthermore, the highlighted regions are generally consistent with clinically relevant BI-RADS ultrasound features, particularly lesion boundaries, irregular shapes, non-circumscribed margins, and heterogeneous internal echogenicity. Similar attention patterns were consistently observed across the BUSI, UDIAT, and independent private datasets, indicating that the proposed model captures diagnostically meaningful imaging characteristics rather than irrelevant background information. These observations further support the interpretability and potential clinical applicability of the proposed method.

**Figure 5:**
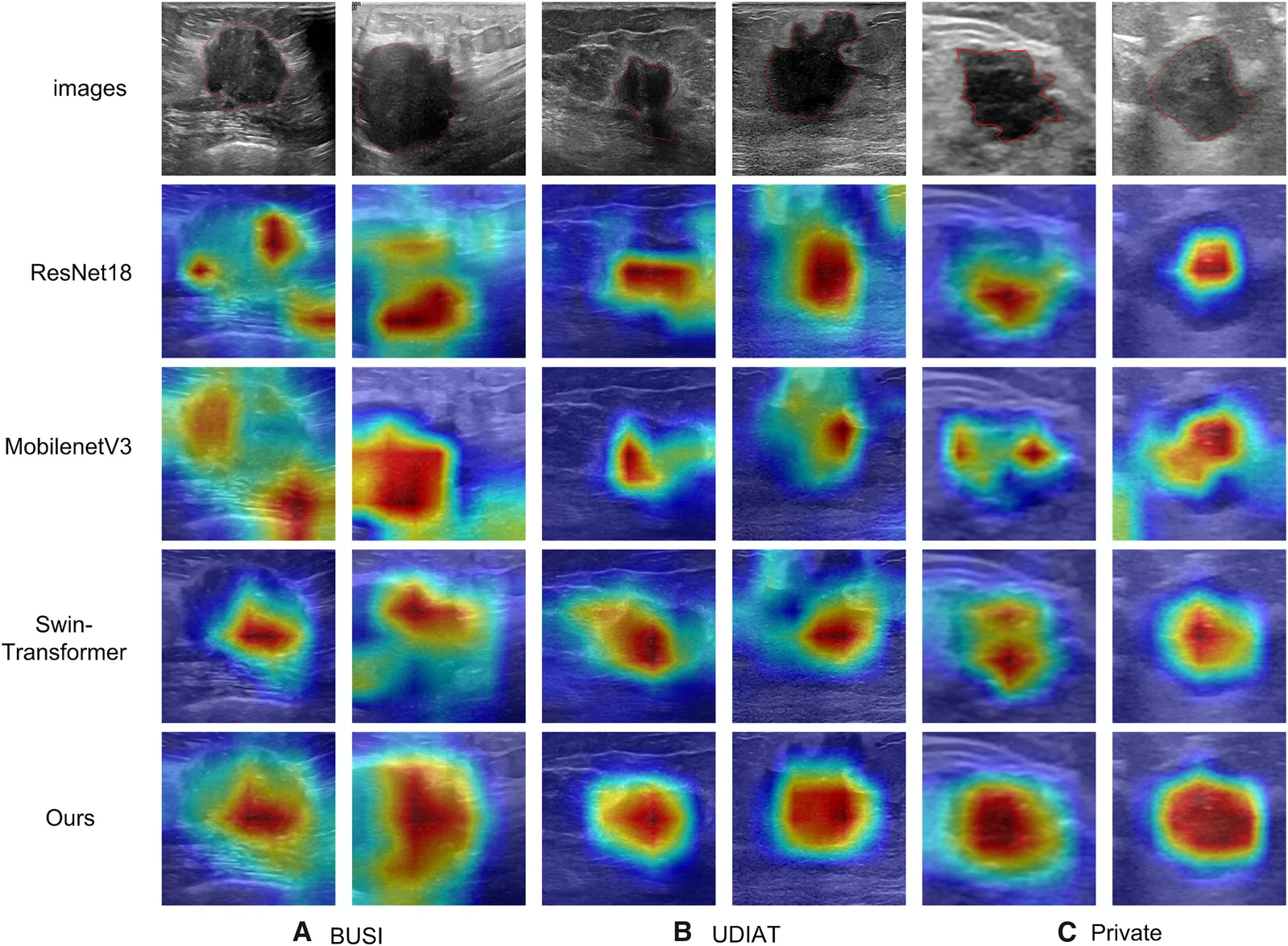
Visualization of features and lesion localization of different models on breast ultrasound images (A) BUSI. (B) UDIAT. (C) Private.

Considering the importance of computational efficiency for practical clinical deployment, we further evaluated the computational cost of the proposed model. As shown in Table 9, the proposed model achieved a favorable balance between computational efficiency and classification performance. It required only 4.12 M parameters and 0.32 G FLOPs, which were substantially lower than most CNN- and Transformer-based baseline models. Meanwhile, the proposed model achieved an inference latency of 7.38 ms and a peak memory consumption of 23.93 MB, demonstrating competitive inference efficiency and modest resource requirements. Although some lightweight models exhibited lower computational costs, they showed inferior classification performance. All latency and memory measurements were conducted under the same hardware and software environment described in the model training section. These results indicate that the proposed model achieves high classification performance with reduced computational burden, supporting its potential application in practical clinical scenarios.

**Table 9:** Computational efficiency comparison of different models

Model	Params (M)	FLOPs (G)	Latency (ms)	Peak Mem. (MB)
DarkNet	41.61	29.73	25.84	523
ShuffleNetV2	2.28	0.15	5.42	14.74
ResNet18	11.69	1.82	8.16	93.38
MobilenetV3	2.54	0.06	5.87	19.26
MobilenetV4	3.82	0.23	6.31	22.84
Swin-Transformer	28.29	4.49	14.28	298.17
Vit	86.57	17.58	23.74	612.35
ConvNeXt	50.22	8.68	18.67	398.71
Ours	4.12	0.32	7.38	23.93

## Discussion

The breast ultrasound image classification model proposed in this study demonstrates significantly superior performance compared with existing CNN- and Transformer-based methods, showing clear advantages in Acc, robustness, and discriminative power. By integrating multi-feature fusion and attention mechanisms, the model effectively enhances the representation of lesion texture and boundary information, maintaining high classification stability even under complex backgrounds and noisy conditions. The use of KD provides two main benefits: it transfers rich soft-label information from the teacher network, enabling the student network to improve classification performance, and, when combined with hard-label training, it mitigates the negative influence of potential errors in the teacher network. The balance between hard-label supervision and KD is controlled by the weighting coefficient α. When α becomes excessively large, the contribution of the distillation loss decreases, which may limit the transfer of informative soft-label knowledge from the teacher network and weaken the generalization ability of the student network. Conversely, an overly small α may increase the dependence on teacher guidance and reduce the contribution of ground-truth supervision. Therefore, an appropriate balance between hard-label supervision and KD is essential for achieving optimal model performance. Experimental results show that the model achieves consistently high Acc while effectively capturing key features of lesion regions. Compared with traditional convolutional networks, the model performs more stably on small-sample, high-noise ultrasound images; compared with pure Transformer models, it achieves a better balance between local detail sensitivity and global semantic modeling.

In addition to its superior classification performance, the proposed model demonstrates favorable computational efficiency. Compared with the major baseline models, it achieves a good balance between diagnostic performance and computational cost. The relatively low computational complexity and memory requirements indicate its potential for deployment in practical clinical environments, providing a promising solution for real-time computer-aided diagnosis of breast ultrasound images. Furthermore, the lightweight architecture, fast inference speed, and modest memory consumption of the proposed model make it well-suited for deployment on modern GPU-equipped clinical workstations. These characteristics are advantageous for real-time computer-aided diagnosis in routine breast ultrasound examinations. Although the integration of the proposed model into the software systems of commercial ultrasound devices has not yet been investigated, its computational efficiency suggests promising potential for future clinical translation following further software integration and prospective validation. Breast ultrasound interpretation is highly dependent on the experience of radiologists, often resulting in variability in diagnostic performance among clinicians with different levels of expertise. The comparison with radiologists of different experience levels demonstrates that the proposed model achieves consistently high diagnostic Acc, suggesting its potential to reduce inter-operator variability by providing objective and consistent diagnostic support. Rather than replacing radiologists, the proposed model is intended to function as an auxiliary diagnostic tool, particularly for less experienced clinicians, thereby improving diagnostic consistency and supporting clinical decision-making in routine breast ultrasound examinations.

Further visualization analysis using various CAM methods provided intuitive insights into the model’s decision-making process, showing that the model’s attention was mainly concentrated within lesion regions. These findings enhance model interpretability and support its potential application in breast ultrasound lesion classification. However, because standardized BI-RADS annotations were unavailable in the evaluated datasets, the correspondence between CAM-highlighted regions and BI-RADS-related imaging characteristics remains qualitative and requires further validation using expert-annotated datasets. Despite the overall high classification performance, a small number of cases were still misclassified. Most of these challenging cases were associated with extremely small lesions, low image contrast, indistinct lesion boundaries, heterogeneous internal echogenicity, or ultrasound artifacts such as speckle noise and posterior acoustic shadowing. In addition, certain benign and malignant lesions exhibited similar morphological characteristics, making accurate discrimination more difficult. These observations suggest that the remaining errors are mainly attributable to the intrinsic complexity of breast ultrasound imaging rather than deficiencies in the proposed network architecture.

Overall, the proposed method achieves an effective balance between classification Acc, computational efficiency, and model interpretability, demonstrating its potential for breast ultrasound image classification. Future work will focus on integrating multi-modal imaging information, incorporating few-shot learning strategies, exploring real-time deployment, and further improving the recognition of challenging cases with ambiguous imaging characteristics to enhance the model’s generalization ability and clinical applicability.

### Limitations of the study

Despite strong performance across multiple datasets, several limitations remain. Quantitative subgroup analysis showed that the model performance decreased in challenging cases. Although the proposed model achieved an overall Acc of 92.64% and an F1-score of 92.37% on the private dataset, the Acc and F1-score decreased to 87.14% and 86.67% for small lesions ≤10 mm, and to 88.10% and 87.72% for low-contrast images. The lowest performance was observed in lesions that were both small and low-contrast. These findings indicate that extremely small lesion size and weak lesion-background contrast remain important sources of diagnostic uncertainty. Additionally, the experiments were conducted primarily on two public datasets and one private dataset, leaving its cross-device adaptability and robustness in real-world clinical settings to be further validated. Moreover, the datasets used in this study did not provide standardized BI-RADS category information, preventing further subgroup analysis for clinically challenging lesions, such as BI-RADS category 4 nodules.

To address these limitations, future work could focus on multiple directions. Multi-center and multi-disease validation should be conducted to comprehensively assess robustness across diverse patient populations, imaging devices, and acquisition conditions. Future studies will also investigate the performance of the proposed model on datasets with standardized BI-RADS annotations to further evaluate its clinical applicability. Integrating multi-modal medical imaging information, such as combining ultrasound with mammography or MRI, could further enhance discriminative ability for complex lesion morphologies and tissue characteristics. Finally, adaptive feature enhancement, online learning, or few-shot learning techniques could be explored to improve robustness in data-scarce scenarios, enhance detection of small or subtle lesions, and facilitate future deployment in real-world clinical environments.

## Resource availability

### Lead contact

Requests for further information and resources should be directed to and will be fulfilled by the lead contact, Tao Hu (sitp6497@126.com).

### Materials availability

This study did not generate new unique reagents.

### Data and code availability

• This paper analyzes existing, publicly available datasets, accessible from the original sources listed in the key resources table. Anonymized private data reported in this paper will be shared by the lead contact upon request.
• Original code has been deposited on GitHub (https://github.com/area6497/UBND) and is publicly accessible as of the publication date.
• Any additional information required to reanalyze the data reported in this paper is available from the lead contact upon request.

## Acknowledgments

This work was supported by the “Pioneer” and “Leading Goose” R&D Program of Zhejiang Province (no. 2023C040309), the Zhejiang Provincial Medical and Health Science and Technology Program Project (no. 2024XY114), and the 10.13039/501100018552Science and Technology Plan Project of Taizhou (no. 26ywb179).

## Author contributions

Conceptualization, T.H., V.Y.W., and D.X.; data curation, T.H., R.R., and Y.D.; formal analysis, T.H. and V.Y.W.; funding acquisition: R.R. and D.X.; investigation, T.H., R.R., Y.D., and D.X.; methodology, T.H. and V.Y.W.; project administration, T.H. and D.X.; resources, Y.D. and D.X.; software, T.H.; supervision: R.R., V.Y.W., and D.X.; validation, V.Y.W. and R.R.; visualization, T.H. and V.Y.W.; writing – original draft, T.H. and V.Y.W.; writing – review and editing, R.R. and D.X.

## Declaration of interests

The authors declare no competing interests.

## STAR★Methods

### Key resources table

**Table:** 

REAGENT or RESOURCE	SOURCE	IDENTIFIER
**Deposited data**		

BUSI	Al-Dhabyani et al.^38^	https://scholar.cu.edu.eg/?q=afahmy/pages/dataset
UDIAT	Yap et al.^39^	https://helward.mmu.ac.uk/STAFF/M.Yap/dataset.php

**Software and algorithms**		

Python	3.8	https://www.python.org/
Torch	1.7.1	https://pytorch.org/
Code for model development	This paper	https://github.com/area6497/UBND

### Experimental model and study participant details

#### Ethics statement

This retrospective study was approved by the Ethics Committee of Zhejiang Cancer Hospital (IRB-2023-125). The requirement for informed consent was waived due to the retrospective nature of the study and the use of fully anonymized data. All procedures were conducted in accordance with the Declaration of Helsinki.

#### Datasets

Two public breast ultrasound image datasets and one private dataset were used to evaluate the performance of the proposed method. The first public dataset is the BUSI dataset,^38^ which includes 437 benign cases, 210 malignant cases, and 133 normal cases, with patients aged between 25 and 75 years. To focus on benign–malignant tumor classification, normal cases were excluded, and only benign and malignant images were retained for subsequent analysis. The second public dataset is the UDIAT dataset,^39^ which consists of 163 well-annotated breast ultrasound images, including 53 malignant cases and 110 benign cases. With precise lesion annotations and representative benign–malignant distributions, this dataset serves as a valuable benchmark for evaluating breast ultrasound–based diagnostic methods.

In addition, a private breast ultrasound dataset retrospectively collected from Zhejiang Cancer Hospital was employed to further evaluate the robustness and generalization capability of the proposed method. The dataset contains 503 B-mode breast ultrasound images, including 260 benign and 243 malignant lesions, all confirmed by pathological examination. Ultrasound examinations were conducted using the following machines: LOGIQ E9 (GE Healthcare), EPIQ7 and iU22 (Philips Medical Systems), and Toshiba-Aplio 500 (Canon Medical Systems). All scans were performed with a linear array transducer with a 5–12 MHz broadband frequency. Trapezoidal imaging mode with expanded field of view was employed for larger lesions that could not be fully displayed within a single frame. For lesions shown in multiple images, all available data were considered. The image resolution ranged from 118 × 62 to 836 × 611 pixels, and the lesion size ranged from 5 mm to 25 mm. Eligible cases were retrospectively selected by experienced breast ultrasound physicians according to predefined inclusion and exclusion criteria. Since this study focused on image-level benign–malignant classification, no additional manual lesion annotation or segmentation was performed. The image-level class labels were established according to the pathological diagnosis, which served as the reference standard. The age distribution of participants in the private dataset is shown in Figure S1. The available clinical and demographic characteristics of the study participants are summarized in Table S1.

Prior to model training, image preprocessing was performed to improve data quality and model robustness. Specifically, a Non-Local Means (NLM) denoising method was applied to suppress speckle noise inherent in ultrasound images while preserving structural details. For the two public datasets (BUSI and UDIAT), the proposed model was evaluated using 5-fold cross-validation. In each fold, data augmentation techniques, including random horizontal and vertical flipping, rotation, color jittering, affine transformation, random cropping, and perspective transformation, were applied exclusively to the training set to expand the training dataset by a factor of five, while the validation set remained untouched throughout the training and evaluation process to prevent potential data leakage. These augmentation strategies were designed to increase data diversity and enhance the generalization ability of the proposed model. Furthermore, the private breast ultrasound dataset was used exclusively as an independent external validation cohort to evaluate the generalizability of the proposed model. The external validation dataset was not involved in model training, data augmentation, or hyperparameter optimization.

### Method details

#### Overall architecture

This study adopted an overall design strategy combining a Teacher–Student KD framework. Input breast ultrasound images are fed into both the pre-trained teacher network and the student network simultaneously. The soft-label generated by the teacher network guide the student network to learn its high-level semantic representations through distillation loss, effectively approximating the recognition performance of the teacher model. The student network uses a CNN as its main backbone, with targeted pruning applied to reduce the number of IB modules. Meanwhile, a Dual-IB structure is introduced to enhance feature representation capability. The front-end of the network employs a standard 3×3 convolution as the input layer, followed by sequential multi-stage feature extraction and structure expansion using modules such as Fused-IB, ExtraDW, and the proposed Dual-IB. This design balances network depth and representation capability. Residual feature aggregation is adopted in the Dual-IB module to facilitate feature reuse and improve gradient propagation during network optimization.

At higher semantic levels, an adaptive hierarchical feature fusion module is introduced between the ConvNeXt module and the last Dual-IB module to fuse low-level and high-level features from different layers. The HF Fusion module can adaptively integrate local and global information based on the content of input features, enabling joint modeling of multi-scale semantics. Finally, the feature maps are processed through global average pooling and a classification layer to produce prediction results. Overall, the network achieves a strong balance between high performance and interpretability, making it suitable for accurate breast ultrasound image classification and auxiliary diagnosis in clinical environments. The network structure proposed in this paper is shown in Figure S2.

#### Dual inverted bottleneck block

The Dual-IB module is designed to simultaneously capture fine-grained local structural information and global texture information under a large receptive field, thereby enhancing the network’s ability to model lesion patterns of different scales in breast ultrasound images. Its structure mainly consists of three components: dual depthwise convolution (DWConv) branches, feature fusion with channel attention enhancement, and residual connections. Firstly, the input feature map X is fed into two depthwise separable convolution branches with different kernel sizes simultaneously: one adopts 3×3 DWConv to extract fine local edges and texture structures; the other uses 5×5 DWConv to obtain a larger receptive field, thus capturing richer contextual texture and morphological information. The operations of the two branches can be expressed as follows:

(Equation 1) $$F_{3\times 3}=DWConv_{3\times 3}\left(X\right)$$

(Equation 2) $$F_{5\times 5}=DWConv_{5\times 5}\left(X\right)$$

Subsequently, the features from the two branches are concatenated along the channel dimension and weighted fused via a channel attention module Squeeze-and-Excitation (SE). The SE module can learn the dependencies between different channels and assign higher weights to task-relevant channels, thereby enhancing key semantic features and making the fused feature representation more discriminative. The fused feature is expressed as follows:

(Equation 3) $$F_{dual}=Concat\left(SE\left(F_{3\times 3}\right),SE\left(F_{5\times 5}\right)\right)$$

Next, a 1 × 1 convolution is employed to compress the channel dimension of the fused features, aligning it with that of the input feature map *X* while reducing computational overhead. Finally, a residual connection is introduced to perform element-wise addition between the transformed features and the input feature map, facilitating feature reuse and improving gradient propagation during network optimization. The final output is expressed as follows:

(Equation 4) Y=Fdual+X

The design of the Dual-IB module balances local details and global semantics simultaneously. It enhances key channel features through an attention mechanism and preserves original information via a residual structure, thereby comprehensively improving the network’s ability to model features of breast lesions across different scales.

#### Hierarchical feature fusion

In this network, low-level features are rich in details but weak in semantics, while high-level features have strong semantics but suffer from detail loss. To address this contradiction, effective fusion of high-level and low-level features becomes a necessary approach. The semantics of high-level features can guide low-level features to filter irrelevant details and highlight key regions, thereby reducing noise interference; meanwhile, the spatial details of low-level features can supplement structural information for high-level features, enabling more accurate localization and boundary description of semantic content. This achieves efficient collaborative representation of low-level and high-level features by preserving high-resolution detailed features while fusing semantic information from deep layers. An adaptive hierarchical feature fusion module is introduced between ConvNeXt and the last Dual-IB module to simultaneously capture local edges and global tissue semantic information. To facilitate feature fusion, the feature map L output by the ConvNeXt module is downsampled by a factor of two.

(Equation 5) $$L_{down}=AvgPool\left(L\right)$$

Spatial attention enhancement is performed on the downsampled feature map.

(Equation 6) $$\hat{L}=\sigma \left({\text{Conv}}_{7\times 7}\left(\left[\text{AvgPool}\left(L_{down}\right),MaxPool\left(L_{down}\right)\right]\right)\right)⊗L_{down}$$

where σ denotes the sigmoid function. Channel attention enhancement is applied to the high-level feature map G:

(Equation 7) $$\hat{G}=\sigma \left(\text{MLP}\left(\text{AvgPool}\left(G\right)\right)+\text{MLP}\left(\text{Maxpool}\left(G\right)\right)\right)⊗G$$

Concatenate the features after channel attention enhancement and spatial attention enhancement:

(Equation 8) $$F_{concat}=Concat\left[\hat{L},\hat{G}\right]$$

The channel fusion via a 1×1 convolution is expressed as follows:

(Equation 9) $$F_{\text{fused}}={\text{ConV}}_{1\times 1}\left(F_{\text{concat}}\right)$$

Nonlinear transformation and residual enhancement can be achieved using an inverted residual MLP (IRMLP). The IRMLP consists of a 3×3 DWConv with a residual connection, along with two linear transformation layers. A GELU nonlinear activation function is applied on the expanded channel dimension. This process is described by the following formula:

(Equation 10) $$\text{IRMLP}\left(F_{\text{fused}}\right)=Conv_{1\times 1}\left(Conv_{1\times 1}\left({DWCon}_{v3\times 3}\left(F_{\text{fused}}\right)+F_{\text{fused}}\right)\right)$$

The final output is expressed as follows:

(Equation 11) $$F_{\text{HE}}=\text{IRMLP}\left(F_{\text{fused}}\right)+F_{\text{fused}}$$

#### Knowledge distillation model

To improve the model Acc, this study introduces a KD strategy to transfer knowledge from a complex teacher network to a student network. By mimicking the output distribution of the teacher network, this method enables the student network to achieve stronger generalization ability on small-scale datasets, leveraging the teacher network’s classification confidence distribution and class relationship information for input samples to guide the student network’s learning process.

During training, the input breast ultrasound images first pass through the teacher network to generate soft -label, and then through the student network to produce prediction results (Student Prediction). The outputs of the two networks are jointly optimized via a distillation loss function to realize knowledge transfer. The Softmax probability distribution output by the teacher network contains relative relationship information between classes, rather than merely classification results. The teacher network adopted in this study is DenseNet121. A temperature parameter T is introduced to adjust the smoothness of the Softmax distribution. The class probability distributions of the teacher network and the student network under temperature T are respectively expressed as follows:

(Equation 12) $${p_{t}}^{T}=softmax\left(\frac{z_{t}}{T}\right),{p_{s}}^{T}=softmax\left(\frac{z_{s}}{T}\right)$$

(Equation 13) $$L_{KD}=T^{2}.KL\left(P_{t}^{T},P_{s}^{T}\right)$$

(Equation 14) $$KL\left({p_{t}}^{T}∥{p_{s}}^{T}\right)=\sum_{i}{p_{t}}^{T}\left(i\right)\log\frac{{p_{t}}^{T}\left(i\right)}{{p_{s}}^{T}\left(i\right)}$$

where KL denotes the Kullback–Leibler divergence between the probability distributions of the teacher and student networks, and *L*_*KD*_ represents the soft-label distillation loss. *z*_*s*_ denotes the output logits of the student network; *z*_*t*_ denotes the output logits of the teacher network; T is the temperature parameter; ${p_{t}}^{T}$ denotes the softened output probability distribution of the teacher network with temperature T; ${p_{s}}^{T}$ denotes the softened output probability distribution of the student network with temperature T.

#### Hybrid loss function

In breast image classification tasks, there exist significant differences in the difficulty of classifying different samples. Traditional cross-entropy loss struggles to enhance the recognition of complex samples. To improve the model’s discriminative capability for hard-to-classify samples, this study introduces a hybrid LMF loss function defined as:

(Equation 15) $$\text{LMFLoss}={\alpha 1}^{*}\text{LDAMLoss}+{\left(1-\alpha 1\right)}^{*}\text{FL}\left(P_{t}\right)$$

(Equation 16) $$LDAMLoss=\frac{1}{N}\sum_{i=1}^{N}w_{y_{i}}\;\log\left(p_{y_{i}}\left(x_{i}\right)\right)$$

(Equation 17) $$\text{FL}\left(p_{t}\right)=-a_{t}{\left(1-p_{t}\right)}^{\gamma }\;\log\left(p_{t}\right)$$

where FL denotes the Focal Loss function, N is the batch size, x_i_ represents the input feature of the i-th sample, y_i_ is the true class index of the i-th sample, $p_{y_{i}}\left(x_{i}\right)$ denotes the probability that the model predicts the i-th sample belongs to its true class y_i_, and $w_{y_{i}}$ is the weight corresponding to the true class y_i_. p is the probability predicted by the model; when the true label is positive, p_t_ = p; when the true label is negative, p_t_ = 1-p. α1 is the weight of the LDAMLoss function, and experimental results show that α1 = 0.5 yields the optimal performance.

The LMF loss function is a weighted combination of the above two loss functions. It leverages Focal Loss to effectively handle hard-to-classify samples and LDAMLoss to enhance feature discrimination, thereby significantly improving classification performance. The final loss function is defined as:

(Equation 18) $$L_{final}=\alpha .\;LMFLoss+\left(1-\alpha \right).L_{KD}$$

where *α* is the balance parameter between the two losses; LMFLoss denotes the hard-label hybrid loss; *L*_*KD*_ denotes the distillation loss.

### Quantification and statistical analysis

To quantitatively assess the performance of benign–malignant breast tumor classification, multiple widely used evaluation metrics were employed to provide a comprehensive analysis of the proposed model. These metrics include accuracy (Acc), precision (Pr), recall (Re), F1-score, receiver operating characteristic (ROC) curve, area under the ROC curve (AUC), confusion matrix, number of parameters (Params), floating-point operations (FLOPs), inference latency (Latency), and peak memory usage (Peak Mem.). Among them, the ROC curve was utilized to illustrate the trade-off between the true positive rate and false positive rate across different decision thresholds, with the area under the ROC curve (AUC) serving as an important indicator of the model’s discriminative capability. In addition, confusion matrix was employed to intuitively visualize the correspondence between predicted labels and ground truth. Params, FLOPs, Latency, and Peak Mem. were used to evaluate the model complexity, computational cost, inference efficiency, and memory consumption, respectively.

#### Model training

Experiments were conducted on a 64-bit Windows 10 operating system, using PyTorch 1.7.1 as the deep learning framework and OpenCV 4.5.5.64 as the image processing library. The hardware environment included an NVIDIA GeForce RTX 3090 graphics card, an Intel(R) Core(TM) i7-12700 processor (2.10 GHz), and 64 GB of memory. The computational efficiency evaluation, including inference latency and peak memory consumption, was performed under the same hardware and software configuration.

During model training, the input image size was uniformly resized to 224×224, with a batch size of 32 and a total of 150 training epochs. The AdamW optimizer was adopted with an initial learning rate of 0.0008 and a weight decay of 1e-4. A Cosine Annealing learning rate scheduling strategy was used, with a minimum learning rate of 1e-6. The distillation temperature (T) was set to 4, 6, and 8, and the weight coefficient α was set to 0.5, 0.6, and 0.7. All experiments were performed with KD enabled to systematically evaluate the impact of different distillation parameters on model performance.

For the BUSI and UDIAT datasets, the teacher model (DenseNet121) and the student model were trained using the same training split within each fold of the five-fold cross-validation. The teacher model was first trained and subsequently used to generate softened probability distributions for supervising the student model. Data augmentation was applied exclusively to the training set, while the corresponding validation set in each fold was used for hyperparameter selection. The independent private breast ultrasound dataset was reserved exclusively for external validation and was not involved in model training, hyperparameter tuning, or model selection.

#### Performance metrics

The performance of the proposed model was comprehensively evaluated using the standard metrics defined in the quantification and statistical analysis section. To further illustrate the discriminative capability of the learned features, t-SNE visualization was first performed on the breast ultrasound datasets (Figure 1), demonstrating the separability of benign and malignant lesions in the learned feature space. Various CAM methods were then employed to visualize the regions contributing to the classification decisions (Figure 2). The confusion matrix on the external validation dataset (Figure 3) was subsequently presented to provide an intuitive overview of the classification results and misclassification patterns. Furthermore, the ROC curves of different models on the private dataset were compared (Figure 4), with the AUC values used to evaluate their discriminative performance. Finally, feature visualization and lesion localization results of different models were compared (Figure 5), highlighting the superior localization capability and interpretability of the proposed model.
