# Supplementary material for: Ultrasound-based breast nodule malignancy diagnosis using CNN enhanced by knowledge distillation
Source: iScience. 2026 Oct 26;29(11):118030. doi: 10.1016/j.isci.2026.118030 (PMC13734998; doi:10.1016/j.isci.2026.118030)
Supplement: Supplementary file 1 [file mmc1.pdf]

iScience, Volume *29*

Supplemental information

Ultrasound-based breast nodule malignancy diagnosis using CNN enhanced by knowledge distillation

Tao Hu, Rongrong Ru, Yingying Ding, Vicky Yang Wang, and Dong Xu

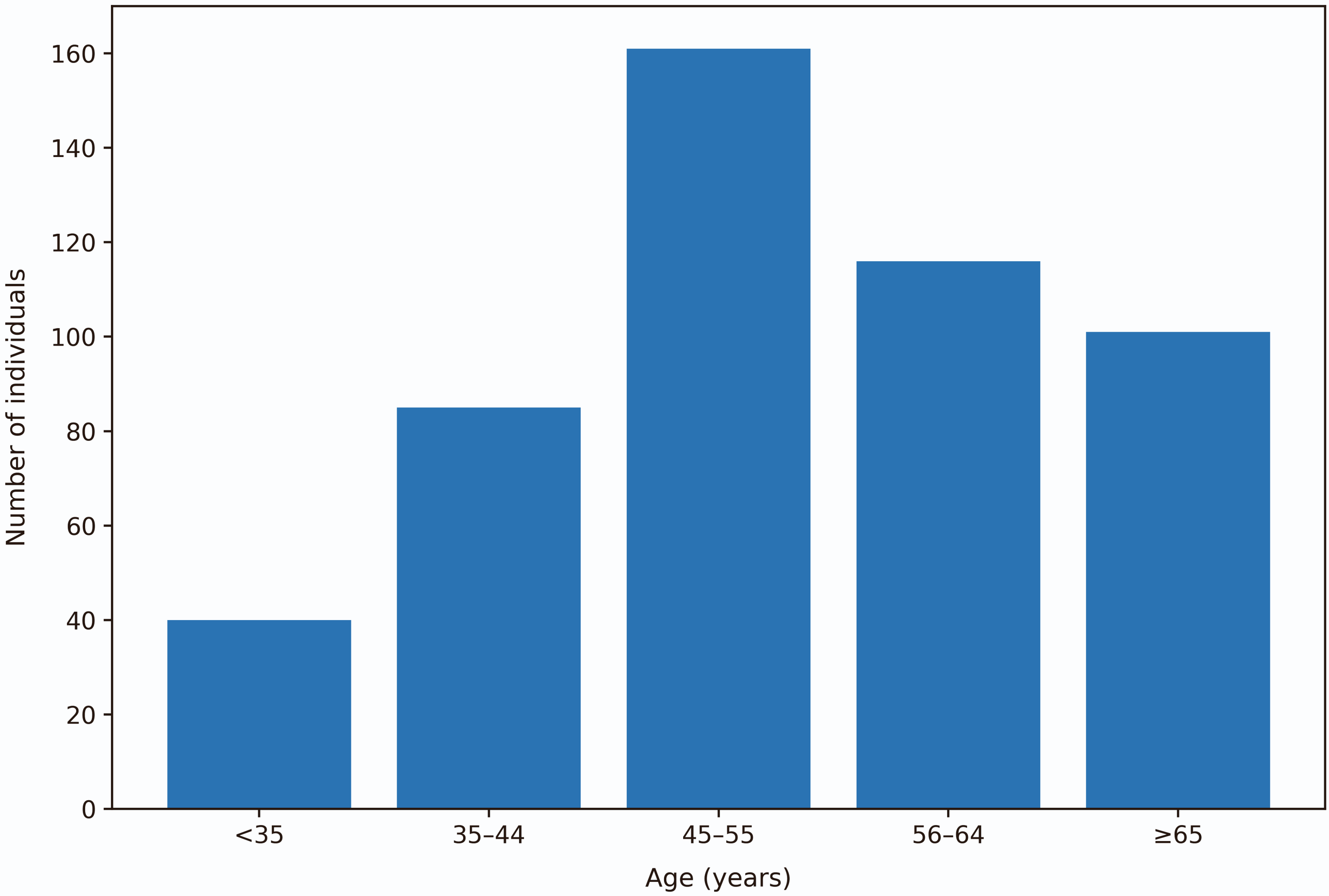

Figure S1. The age distribution of participants in the private dataset

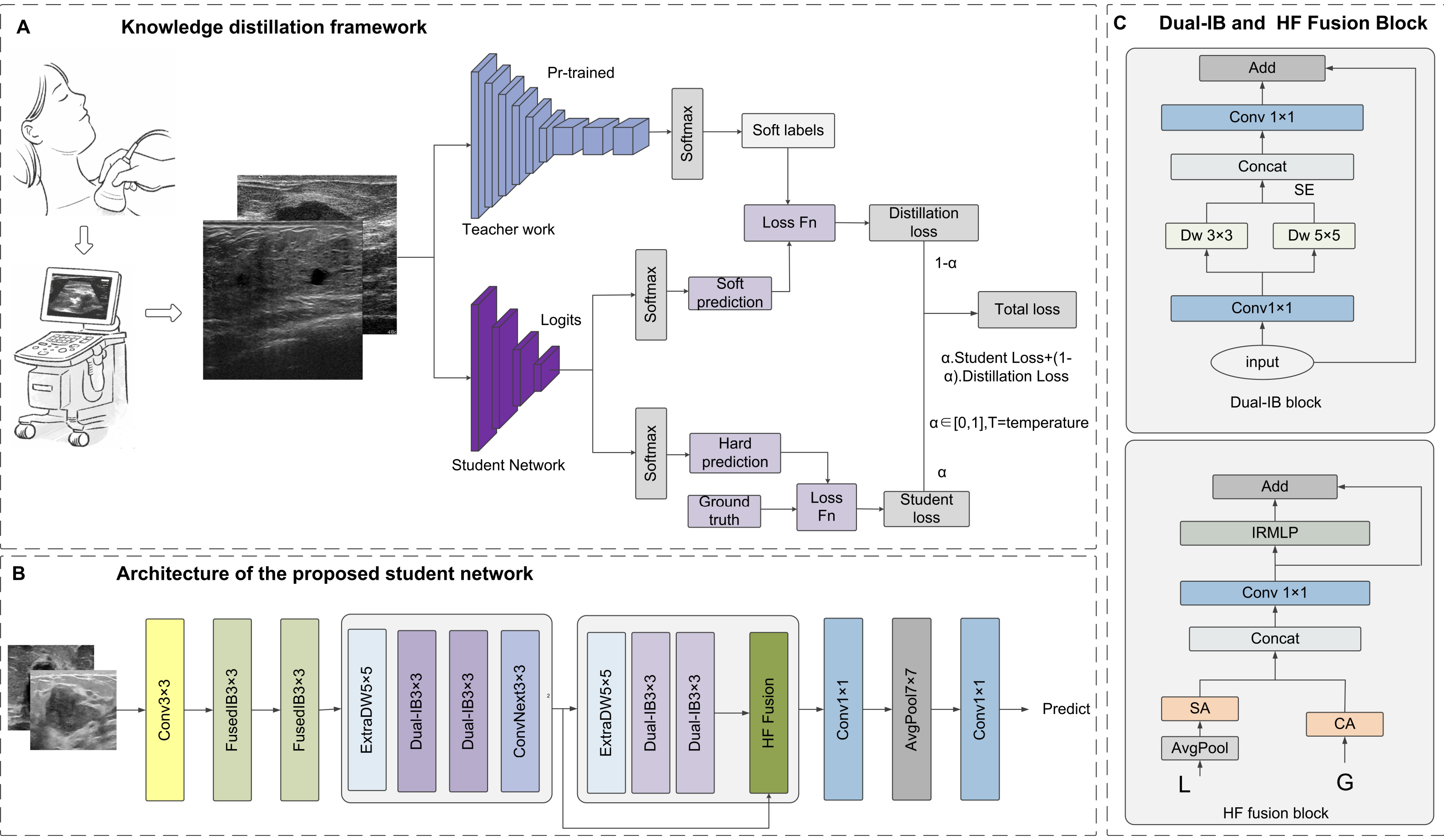

Figure S2. Overall architecture of the proposed network. (A) Knowledge distillation framework, where a pre-trained teacher guides the student model by providing soft targets with temperature T. (B) Overall architecture of the proposed student network, consisting of efficient convolutional, attention, and fusion blocks, designed to enhance feature learning. (C) Detailed structures of the proposed Dual-IB block and hierarchical feature (HF) fusion block

Table S1. Clinical and demographic characteristics of the study participants

Characteristic	BUSI	UDIAT	Private dataset
Benign, n (%)	437 (67.5%)	110 (67.5%)	260 (51.7%)
Malignant, n (%)	210 (32.5%)	53 (32.5%)	243 (48.3%)
Age, years	25-75	NR	22-79
Female, n (%)	647 (100%)	163 (100%)	503 (100%)
Ancestry	NR	NR	East Asian
Race	NR	NR	Asian
Ethnicity	NR	NR	Han Chinese

Abbreviations: n, number of participants; NR, not reported. Demographic information unavailable from the original public datasets or associated publications is indicated as NR.
